# Feature Point Tracking-Based Localization of Colon Capsule Endoscope

**DOI:** 10.3390/diagnostics11020193

**Published:** 2021-01-28

**Authors:** Jürgen Herp, Ulrik Deding, Maria M. Buijs, Rasmus Kroijer, Gunnar Baatrup, Esmaeil S. Nadimi

**Affiliations:** 1 Faculty of Engineering, Applied Artificial Intelligence and Data Science, Maersk Mc-Kinney Moller Institute, University of Southern Denmark, 5230 Odense, Denmark; esi@mmmi.sdu.dk; 2 Institute of Clinical Research, University of Southern Denmark, 5230 Odense, Denmark; Ulrik.Deding@rsyd.dk (U.D.); maria.magdalena.buijs@rsyd.dk (M.M.B.); Rasmus.Kroeijer@rsyd.dk (R.K.); gunnar.baatrup@rsyd.dk (G.B.); 3 Department of Surgery, Odense University Hospital, 5700 Svendborg, Denmark

**Keywords:** capsule endoscopy, feature points tracking, localization, large bowel

## Abstract

In large bowel investigations using endoscopic capsules and upon detection of significant findings, physicians require the location of those findings for a follow-up therapeutic colonoscopy. To cater to this need, we propose a model based on tracking feature points in consecutive frames of videos retrieved from colon capsule endoscopy investigations. By locally approximating the colon as a cylinder, we obtained both the displacement and the orientation of the capsule using geometrical assumptions and by setting priors on both physical properties of the intestine and the image sample frequency of the endoscopic capsule. Our proposed model tracks a colon capsule endoscope through the large intestine for different prior selections. A discussion on validating the findings in terms of intra and inter capsule and expert panel validation is provided. The performance of the model is evaluated based on the average difference in multiple reconstructed capsule’s paths through the large intestine. The path difference averaged over all videos was as low as 4±0.7 cm, with min and max error corresponding to 1.2 and 6.0 cm, respectively. The inter comparison addresses frame classification for the rectum, descending and sigmoid, splenic flexure, transverse, hepatic, and ascending, with an average accuracy of 86%.

## 1. Introduction

Wireless capsule endoscopy may be a useful supplement to colonoscopy for non-invasive diagnostics of the digestive system. It can either supplement incomplete colonoscopy or be a useful diagnostic test investigating the need for a therapeutic colonoscopy or surgery. The invasive nature of colonoscopy may cause discomfort and unwanted side-effects which might negatively affect acceptability and cause diagnostic delay; performing a capsule study first might circumvent some of the issues. Severe complications after colonoscopy are infrequent in screening programs, but, in the high number of investigations with a relatively low frequency of positive findings, it causes concerns. Colon capsule endoscopy (CCE) is especially beneficial in patients with previous incomplete attempted colonoscopies. Knowing the location of the capsule, even an incomplete CCE may complete an incomplete colonoscopy investigation as long as it captures the most proximal point of the previous conducted attempt. Deding et al. [1] underlined this clinical importance of a localization algorithm that is able to estimate the whereabouts of the capsule.

**Figure 1:**
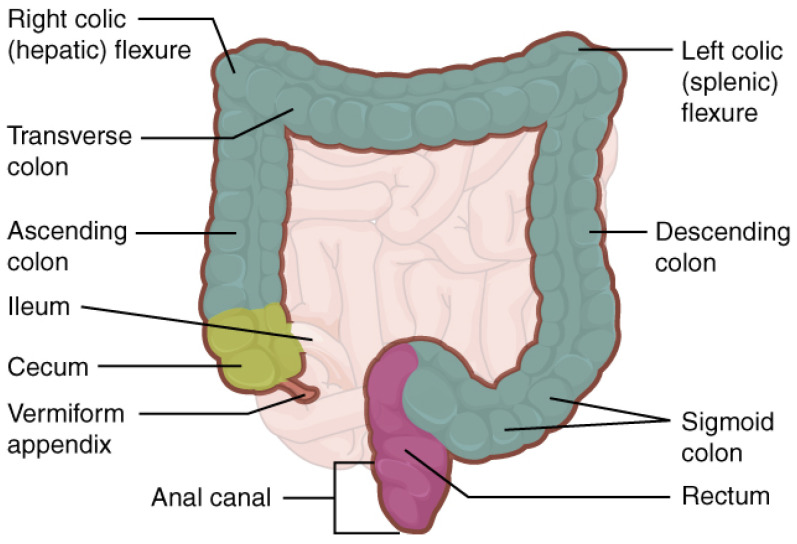
Illustration of the large intestine. Taken from *Anatomy and Physiology*, OpenStax (Licensed: CC-BY).

The large intestine runs from the ileocecal valve to the anus (See Figure 1). It frames the small intestine, despite being half the length. The diameter varies several folds from the wide cecum to the narrow sigmoid and rectum. The length, diameter, and position of individual sections vary significantly from person to person.

When the capsule moves through the intestine, it records frames that are sent wirelessly to a mobile receiver [2]. The portability of the systems allows patients to be free to pursue their regular daily routine without being confined to a medical facility. Studies have shown that the capsule-based endoscopy aids in detecting an increasing number of medically relevant features, e.g., intestinal bleeding, ulcers, vascular lesions, inflammatory diseases, polyps, tumors, and cancers [3,4,5,6,7,8,9,10].

The large bowel camera capsule technology might be more sensitive and specific in polyp detection than colonoscopy. Polyp miss rate of colonoscopy is estimated to be 22% and polyps flat in nature can be particularly challenging to identify [11,12]. In a population of 253 colorectal cancer screening individuals, CCE proved more sensitive to polyps than colonoscopy in complete investigations [13]. On the other hand, the capsule technology is lacking the therapeutic capability when early pathologies, suitable for endoscopic resection, are demonstrated. Therefore, positive CCE investigations are often followed by a therapeutic colonoscopy. An accurate indication of the position of pathologies would be of help to the succeeding endoscopist and would potentially reduce time, resources, and risk of complications for the patient. Even though capsule systems provide a non-invasive and portable monitoring system, the inability to estimate or track its whereabouts requires a physician or trained staff with extensive domain knowledge and practice. This work proposes a tool to aid the process of localizing and tracking the capsule inside the human body. More precisely, this study proposes a model that can identify the two flexures, ascending-, transverse-, and descending-colon parts of the large intestine. In many cases, polyp identified by CCE is not found in the succeeding colonoscopy [13,14], and, thus, such information is of clinical importance to the physicians, as it narrows down the location of the disease, reducing the time and ambiguity of following therapeutic endoscopies and increased chance of successful identification of polyps.

Numerous publication bare witness to the attempts of capsule localization and tracking [15,16,17,18,19,20,21], of which selective ones were summarized by Bianchi et al. [22] and Mateen et al. [23] and are outlined below. Radio frequency (RF) localization and other external approaches, such as magnetic field based localization, rely on the modeling of the tissue between transmitter and receivers and are sensitive to the position of the receivers. The localization of the endoscopy capsule is done in the three-dimensional space of the human body, using RF triangulation from external sensor arrays in combination with time-to-arrival estimations. Studies based on simulations and in vitro experiments have shown that RF localization can be performed and can theoretically achieve precise and accurate results [24]. Isolated in vivo studies have addressed RF localization as well. Nadimi et al. [25] investigated, through trial programs on a live pig, the effective complex permittivity in the gastrointestinal track organs. As a result, updated models for the effective complex permittivity of multilayer non-homogeneous medium have been proposed [26,27]. Recent results from magnetic field localization have experimentally shown to be accurate [18], but implementations are still in their early stages. Development in image processing and deep learning have provided another framework for localization of the endoscopy capsule. It has been demonstrated that, based on geometrical models, pure visual aided localization can be performed in vitro [28,29,30,31,32]. In particular Wahid et al. [19] and Bao et al. [20] provided a simple geometrical approximation to the colon.

One disadvantage of the tracking systems is the wide variation of the large bowel position within the body and its anatomy. Its length, position, movability, and diameter cannot be judged from outside and the external monitored localization might be very difficult to convert into a colonic position usable to the endoscopist. It does thus not come as a surprise that reconstructions of segments or the entire colon is a challenging task. As of the time of writing, the authors are not aware of any published work on a full reconstruction of the large intestine (or segments thereof) based on capsule endoscopy videos in variable anatomy and colon environment, in any of the above frameworks.

The working hypothesis under which this work is conducted has been that an internal picture-based tracking of camera capsules can be easily converted to useful information towards the following endoscopic investigation. Given that external approaches still have to be implemented in real life case studies, and visual based approaches show promising results, the following work demonstrates and supports that visual-based approaches can provide the localization of an endoscopy capsule. In this work, we combine the experimental work into a framework that can fully reconstruct the large intestine. To this end, we assume that the colon properties as well as external factors can be approximated by prior distribution for both the large intestine’s radius and the capsule’s sample frequency. We extend the work of Wahid et al. [19] and Bao et al. [20] to estimate the most likely movement parameters in contrast to ensemble averages and use data from patient’s capsule investigations. Furthermore, we address the information content in each frame by including a measure of cleanliness into the model in order to allow for full colon reconstructions.

## 2. Methodology

The PillCam™ COLON2 system includes the PillCam™ Capsule, a receiver belt, and RAPID™ software. The endoscopy capsule captures images with enhanced optics supported by three lenses. To cover most of the tissue, the camera is equipped with two camera heads that each provide η=172° field of view for a total of 344° coverage. Frames are automatically recorded with varying frequencies, 4–35 Hz.

As the endoscopy capsule captures frames on short time intervals and moves relatively slow compared to the sample frequency, this means that individual frames can contain the same or similar information. This information aids in identifying conditions in the bowel when evaluated by a human expert, such as detecting and tracking of internal bleeding, polyp identification, or assessing the cleanliness of the intestine. However, this relies on the skill of the experts involved and performance may vary [13]. Assuming the shared information across frames are quantifiable, consecutive frames are used to estimate the relative movement of the endoscopy capsule. The proposed model is composed of two parts: (1) a model for the shape of the intestine; and (2) converting recorded video information into a movement estimator for the endoscopy capsule. Both are explained in greater details below.

### 2.1. Model for the Shape of the Intestine

For the time being, let *P* and *Q* be two different points in the intestine. As long as these points are in the field-of-view of the camera head, *P* and *Q* will be recorded in the frames. These points are referred to as feature points.When the endoscopy capsule is moving relative to *P* and *Q*, it will result in mapping the points into feature points in different locations on the frame. Tracking the changes in feature points gives an estimation of the endoscopy capsule’s displacement. However, to do so, it is necessary to provide a model describing the shape of the intestine.

**Figure 2:**
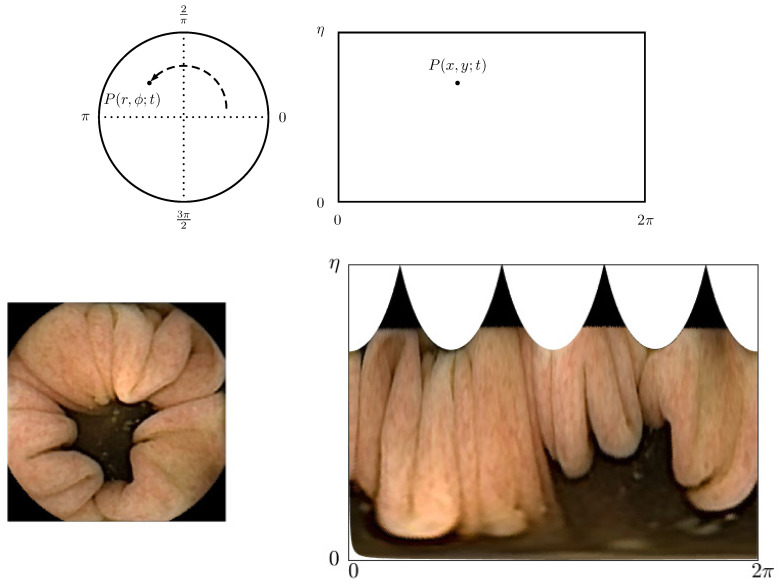
(**Top**) From left to right: Schematic drawing of the frame captured on the CMOS in polar coordinates and transformed frame into Cartesian coordinates. (**Bottm**) From left to right: Actual frame as captured by the CMOS and actual frame as seen when transformed into Cartesian coordinates.

When looking at a single frame, as shown in the lower left corner of Figure 2, one observes that the frame consists of two regions:

1. Dark center region: When the endoscopy capsule is aligned with the intestine, each frame comprises an underexposed area in the center. This area is not illuminated by the light-emitting diode (LED) flash and thus is outside the view range of the camera head.
2. Tissue: The colored tissue forms the wall of the intestine.

This can be said for the majority of the frames, except for frames of poor visual quality and when the camera head is facing only tissue. Thinking of the intestine as a tunnel-shaped object that coils through the human body, with properties as described in Points 1 and 2, a three-dimensional geometric shape could be used as an approximation to its form. For all practical purposes, the proposed model approximates the intestine locally, as a cylinder of radius *R*. This approach was introduced by Wahid et al. [19] and Bao et al. [20]. This framework is extended to include distribution based estimations of the involved parameters, and a post-processing step is added that accounts for the variability in the quality of frames. The extension into the destitution-based estimation, and there-through the most similar parameter value, is a necessity as pure ensemble means can be skewed when *P* and *Q* are errorlessly match between consecutive frames.

**Figure 3:**
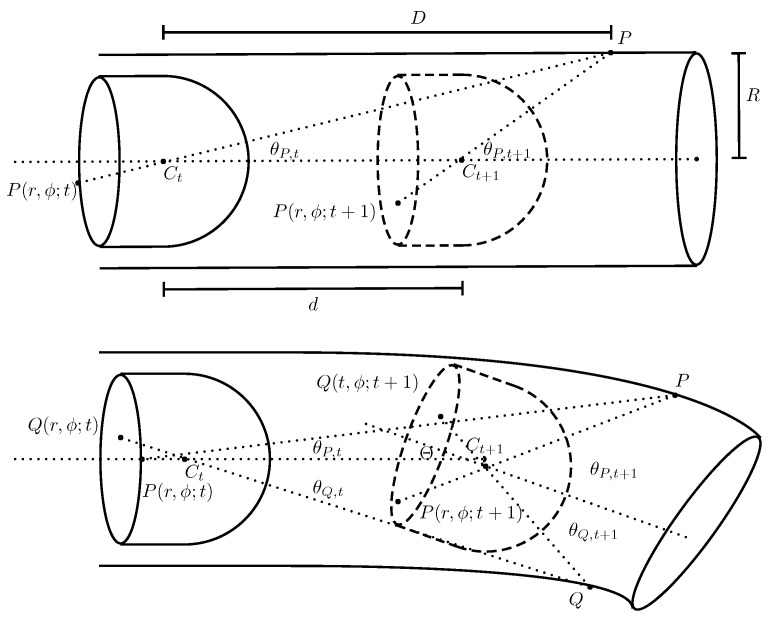
(**Top**) Illustration of a camera head in a cylinder at time *t* and later at time t+1 moved by a distance *d*; and (**Bottom**) illustration of a camera head in a cylinder at time *t* and later at time t+1 but with the cylinder symmetry axis inclined by Θ. Drawing not to scale.

Figure 3 (top) shows a camera head in a cylindrical tube.

A point *P*, at distance *D* from the center point $C_{t}$ of the camera head, is recorded as P(r,ϕ;t) on the CMOS forming the frame at time *t*. Any *P* and its representation on the CMOS form a line that intersects $C_{t}$ with an angle,

(1) $$\begin{array}{c} \theta_{P,t}=\tan^{-1}\left(\frac{R}{D}\right). \end{array}$$

The next sampled frame is taken after the camera head has moved a distance *d*. The same point *P* is now represented by P(r,ϕ;t+1), and its corresponding angle is given by:

(2) $$\begin{array}{c} \theta_{P,t+1}=\tan^{-1}\left(\frac{R}{D-d}\right). \end{array}$$

Solving for *d* in Equation (2) and substituting into Equation (1) gives the distance traveled between times *t* and t+1 as a function of the intestine geometry, namely the radius *R*, and the angles for the projection of *P* at these times:

(3) $$\begin{array}{c} d_{P,t+1}=\frac{R}{\tan(\theta_{P,t+1})}\left(\frac{\tan(\theta_{P,t+1})}{\tan(\theta_{P,t})}-1\right). \end{array}$$

Equation (3) holds true for all *P* in the field-of-view of the camera head. However, it is impossible to localize and track all recorded points, i.e., all pixels of the frame. In this work, feature points are utilized to localize and track information across frames. A feature point is a distinct point of interest in a frame (e.g., edge, corner, blob, or ridge). The task is to find and track corresponding feature points between two frames. There are different approaches for extracting and matching feature points; an implementation of SURF (Speeded-Up Robust Features [33]) was selected as a trade-off between computational complexity and accuracy in identifying and matching feature points. The detected and matched feature points are further filtered for erroneous matching. This is achieved by a random sample consensus (RANSAC) approach [34]. Here, the algorithm takes all matched points and proposes a model for the majority of the feature points. Feature points deviating from that model are considered outliers and removed from the final set of feature points.

Thus far, feature points are said to be recorded on the CMOS of an endoscopy capsule. For computational convenience, the feature points are mapped into a Cartesian coordinate system, as shown in Figure 2, such that x=Lϕ/2π, where *L* is the number of pixels of the frame length and y=r. This allows estimation of the displacements in a linear manner rather than based on arcs in polar coordinates. Figure 2 (bottom) shows how this looks in the case of a single frame. In this coordinate system, the angle $\theta_{P,t}$ is estimated as:

(4) $$\begin{array}{cc} \theta_{P,t} & =\tan^{-1}\left(\frac{r_{P,t}}{R}\tan\left(\eta \right)\right) \end{array}$$

(5) $$\begin{array}{cc} \; & \approx \left(\frac{\eta }{H}\right)y_{P,t}, \end{array}$$

with *H* being the number of pixels of the frame hight.

### 2.2. Capsule Endoscopy Movement Estimation

From now on, this work addresses the relative movement of the endoscopy capsule. The movement estimation, both displacement and orientation, is treated as one-step-ahead predictions. Given the estimates $\theta_{P,t}$ and $\theta_{P,t+1}$ for the case shown in Figure 3 (top), the most likely speed, $v_{t+1}$, of the endoscopy capsule is calculated by evaluating the ensemble distribution, P(·), of displacements $d_{P,t+1}$ multiplied with the sample frequency $f_{t+1}$:

(6) $$\begin{array}{c} v_{t+1}\leftarrow \underset{\forall P}{arg max}P\left(f_{t+1}d_{P,t+1}\right). \end{array}$$

Further, the most likely displacement $\Delta x_{P,t+1}=x_{P,t+1}-x_{P,t}$ in the Cartesian coordinate system is used for an estimation of the roll, $\gamma_{t+1}$, of the endoscopy capsule:

(7) $$\begin{array}{c} \gamma_{t+1}\leftarrow \underset{\forall P}{arg max}P\left(\Delta x_{P,t+1}\right). \end{array}$$

To account for possible tilt of the camera head, the setup, as shown in Figure 3 (bottom) is considered. The camera head at t+1 is inclined by an angle Θ with respect to the alignment of its position at *t*. Depending on the direction in which the endoscopy capsule is facing, the change in angle for any two points in the intestine, *P* and *Q*, is written as $\delta \theta_{P,t+1}=\theta_{P,t+1}-\theta_{P,t}$ and $\delta \theta_{Q,t+1}=\theta_{Q,t+1}-\theta_{Q,t}$. In the case of two opposite points, as shown in Figure 3, the change in angle is larger for *P* than for *Q*. The magnitude of the tilt for a pair of feature points is defined as:

(8) $$\begin{array}{ccc} \Omega =\frac{\delta \theta_{P,t+1}-\delta \theta_{Q,t+1}}{\max(\delta \theta_{P,t+1},\delta \theta_{Q,t+1})}, & \; & \forall P\neq Q. \end{array}$$

The final estimated most likely magnitude of the tilt is given by the distribution over Equation (8):

(9) $$\begin{array}{c} \Omega_{t+1}\leftarrow \underset{\Omega }{arg max}P\left(\frac{\delta \theta_{P,t+1}-\delta \theta_{Q,t+1}}{\max(\delta \theta_{P,t+1},\delta \theta_{Q,t+1})}\right),\\ \;\forall \left\{P,Q|P\left(\frac{\delta \theta_{P,t+1}-\delta \theta_{Q,t+1}}{\max(\delta \theta_{P,t+1},\delta \theta_{Q,t+1})}\geq \xi \right),P\neq Q\right\}, \end{array}$$

where ξ∈[0,1] is the amount of the cumulative distribution of Equation (8) under consideration.

After having estimated $\Omega_{t+1}$, as defined in Equation (9), the associated most likely direction of the tilt is estimated as:

(10) $$\begin{array}{c} \omega_{t+1}\leftarrow \underset{\phi }{arg max}P\left(\phi_{P,t+1}\right),\\ \;\forall \left\{P,Q|P\left(\frac{\delta \theta_{P,t+1}-\delta \theta_{Q,t+1}}{\max(\delta \theta_{P,t+1},\delta \theta_{Q,t+1})}\geq \xi \right),P\neq Q\right\}. \end{array}$$

The information obtained from Equations (9) and (10) is separated into the endoscopy capsule’s pitch and yaw angles:

(11) $$\begin{array}{cc} \alpha_{t+1} & =\Omega_{t+1}\cos\left(\omega_{t+1}\right) \end{array}$$

(12) $$\begin{array}{cc} \beta_{t+1} & =\Omega_{t+1}\sin\left(\omega_{t+1}\right). \end{array}$$

The above-mentioned orientation estimation is based on the yaw, pan, and tilt of the camera’s reference frame. For an external observer, this is transformed into the relative movement of the endoscopy capsule, with respect to a reference point in the observer’s coordinate system, by using a rotation matrix. Let $s_{t}={\left[s_{x},s_{y},s_{z}\right]}^{⊤}$ be a unit vector in the observer’s coordinate system with α,β,γ spanning the vector space. For any consecutive estimations of α, β, and γ, the orientation is then given by:

(13) $$\begin{array}{cc} s_{t+1} & =R_{t+1}^{\mathrm{orientation}}s_{t}, \end{array}$$

where the orientation matrix can be updated as follows:

(14) $$\begin{array}{c} R_{t+1}^{\mathrm{orientation}}=R_{t}^{\mathrm{orientation}}R_{x,t+1}R_{y,t+1}R_{z,t+1}{\left(R_{t}^{\mathrm{orientation}}\right)}^{-1} \end{array}$$

with standard individual rotation matrices:

(15) $$\begin{array}{cc} R_{x,t+1} & =\left[\begin{array}{ccc} 1 & 0 & 0\\ 0 & \cos(\gamma_{t+1}) & -\sin(\gamma_{t+1})\\ 0 & \sin(\gamma_{t+1}) & \cos(\gamma_{t+1}) \end{array}\right], \end{array}$$

(16) $$\begin{array}{cc} R_{y,t+1} & =\left[\begin{array}{ccc} \cos(\beta_{t+1}) & 0 & \sin(\beta_{t+1})\\ 0 & 1 & 0\\ -\sin(\beta_{t+1}) & 0 & \cos(\beta_{t+1}) \end{array}\right], \end{array}$$

(17) $$\begin{array}{cc} R_{z,t+1} & =\left[\begin{array}{ccc} \cos(\alpha_{t+1}) & -\sin(\alpha_{t+1}) & 0\\ \sin(\alpha_{t+1}) & \cos(\alpha_{t+1}) & 0\\ 0 & 0 & 1 \end{array}\right]. \end{array}$$

Given the orientation of the endoscopy capsule and its estimated speed, the tracking of the endoscopy capsule is given by a vector representation in the observer’s frame of reference:

(18) $$\begin{array}{cc} t_{t+1} & =T_{t+1}t_{t} \end{array}$$

(19) $$\begin{array}{cc} \left[\begin{array}{c} x_{t+1}\\ y_{t+1}\\ z_{t+1}\\ s_{t+1} \end{array}\right] & =\left[\begin{array}{cccccc} \; & \; & & \; & & \\ \; & I & \; & v_{t+1}f_{t+1}I & \\ \; & \; & & \; & & \\ \; & \; & & \; & & \\ \; & 0 & \; & R_{t+1}^{\mathrm{orientation}} & \\ \; & \; & & \; & & \end{array}\right]\left[\begin{array}{c} x_{t}\\ y_{t}\\ z_{t}\\ s_{t} \end{array}\right], \end{array}$$

where $t_{t}$ and $t_{t+1}$ are the initial and predicted tracking vectors, T is the matrix mapping from one vector to the other, and $v_{t+1}$ is given by Equation (6).

### 2.3. Post-Processing of Frame Cleanliness

Depending on the quality of the video and ambient conditions in the intestine, it might be necessary to post-process the estimated movement before progressing to the next time stamps. For that, we distinguish between two assessments, one based on the number of feature points in a frame and one based on the cleanliness of a frame. A frame is defined to be of unacceptable quality if the number of matched feature points fall under a threshold. For this study, a threshold of 20 feature points was chosen. Ambient conditions in the intestine are assessed by the cleanliness. The reasoning is best explained by an example: imagine the camera head passes a section of the intestine covered in fecal matter. In this case, no features associated with the intestine are available; thus, the corresponding movement update should account for this. Assessing the cleansing quality has been studied previously [35,36,37,38]. In the following, the cleanliness assessment and the post-processing step are outlined.

For the assessment of the cleanliness, a support vector machine (SVM) classification algorithm based on experts’ input is utilized to contract an assessment model. The SVM is used to determine the cleanliness of individual pixels in a frame, classifying them as either clean or dirty. The frame cleanliness is then determined based on the number of clean and dirty pixels. Pixels that were over- or underexposed are omitted in the assessment. According to Buijs et al. [36], the cleanliness level of a frame at time t+1, $L_{t+1}$, is classified as follows:

(20) $$\begin{array}{c} L_{t+1}=\left\{\begin{array}{cc} 0 & \mathrm{Unacceptable}\\ 1 & \mathrm{Poor}\\ 2 & \mathrm{Fair}\\ 3 & \mathrm{Good} \end{array}\right., \end{array}$$

where for the remainder of this work $L_{t+1}=1,2,3$ are considered to be of adequate quality in order to perform estimations of the movement parameters. In the case of an insufficient frame, the movement update, as described by Equation (18), is changed to estimate the movement from the previous tracking matrix $T_{t}$, penalized by additive noise on the previous estimated parameters, such that:

(21) $$\begin{array}{c} t_{t+1}=\left\{\begin{array}{cc} \left(T_{t}\left(h+\epsilon_{i}\right)\right)t_{t} & \mathrm{if}\;L_{t+1}=0\;\mathrm{or}\;\mathrm{FP}<20\\ T_{t+1}t_{t} & \mathrm{else}\; \end{array}\right., \end{array}$$

where FP is the number of matched feature points, $T_{t}\left(h+\epsilon_{i}\right)$ is a function of *h* and $\epsilon_{i}$, and $\epsilon_{i}∼N\left(0,\sigma_{h}^{2}\right)$ are additive Gaussian distributed noise components for $h=v_{t},f_{t},\alpha_{t},\beta_{t},\gamma_{t}$, with zero mean and variance:

(22) $$\begin{array}{ccc} \sigma_{h}^{2}=\frac{1}{t}\sum_{i=1}^{t}{\left(h_{i}-E\left[h_{\left[1,t\right]}\right]\right)}^{2} & \; & ,h=\alpha ,\beta ,\gamma . \end{array}$$

The expected values in Equation (22) are the mean values of the previous *t* estimated *v*, *f*, α, β, and γ.

## 3. Results

This section explores the experimental set-up, evaluating the proposed model addressing endoscopy capsule videos. The model presented in this work does not have a standardized ground truth validation yet, because it is providing the first attempt at solving the problem of tracking the endoscopy capsule through the intestine. As of the time of writing, the authors are likewise not aware of any ground truth validated approach. This is attributed to the lack of control of the endoscopy capsule after it is swallowed by a patient. However, different studies under controlled environment and simulations have been performed, showing that high accuracy is achieved when estimation translation and rotation based on feature point extraction and matching [19,20,21]. These studies support the idea of the proposed model for capsule videos.

### 3.1. Data and Parameter Selection

This study is comprised of 42 patients, as representative of the study by Kobaek-Larsen et al. [13] (P=84 videos), where each frame is converted to gray-scale. The endoscopy capsule, as a commercial product, only provides access through dedicated software. The RAPID™ software is used to extract videos into a common file format. This comes at the cost of loosing unique time stamps as the exact sampling at variable frequencies is lost. The software compresses the recorded video to fixed frames per seconds. To account for that loss of information, two statistical driven scenarios are considered: (i) the sample frequencies are random and drawn from a continuous uniform distribution, $f_{t+1}∼U\left(a,b\right)$ on the interval [a,b]; or (ii) sample frequencies are truncated Gaussian [39,40] distributed, with mean frequency $\mu_{f}$, standard deviation $\sigma_{f}$, and bound [a,b].

The prior selection is based on the studies by Gilroy et al. [41] and Ohgo et al. [42], where the average diameter of the large intestine is reported to be $\mu_{2R}=4.6$ cm, with standard deviation $\sigma_{2R}=0.7$ cm, on the interval $\left[a_{2R}=2.5,b_{2R}=9\right]$ cm. Following the idea of drawing sample frequencies, the intestine diameter, $2R_{t+1}$, is drawn from either a uniform distribution U(2.5,9) or a truncated Gaussian distribution with $\mu_{2R}$, $\sigma_{2R}$, $a_{2R}$, and $b_{2R}$. For Equations (9) and (10), ξ=0.25 has shown to be suitable for all practical purposes.

**Figure 4:**
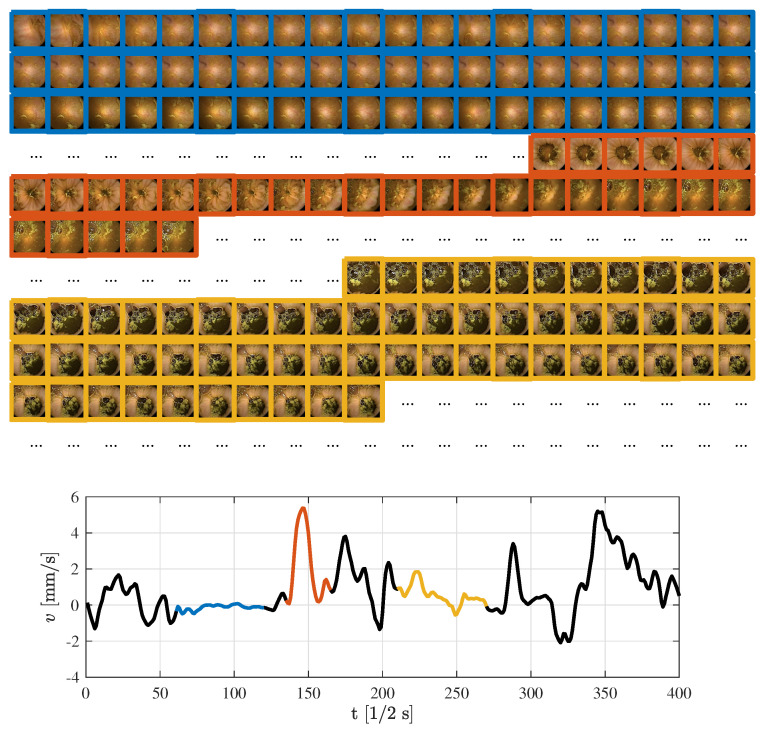
Selected examples of speed estimations based on a fixed sample frequency of 2 Hz and corresponding frames.

The model is illustrated on series of 400 frames sampled at 2 Hz, with intestine radius 5 cm. Figure 4 gives a more intuitive understanding of patterns encountered in an endoscopy capsule video. Here, examples on a stationary case (blue), fast movement (orange), and slow movement (yellow) are given. The first sequence of frames, marked in blue, produce stationary behavior of the endoscopy capsule. This is supported by the corresponding frames above the speed graph, facing the same part of the intestine wall with small or no variations. The next sequence, highlighted in orange, shows a sudden burst of movement.

### 3.2. Results of 42 Patient Study

For simplicity and comparison to colonoscopy, the path taken by the endoscopy capsule is reconstructed from the anus. As shown below, selecting the anus as the origin of the localization and manually choosing the direction have a significant impact on the characteristics of the estimated paths.

**Figure 5:**
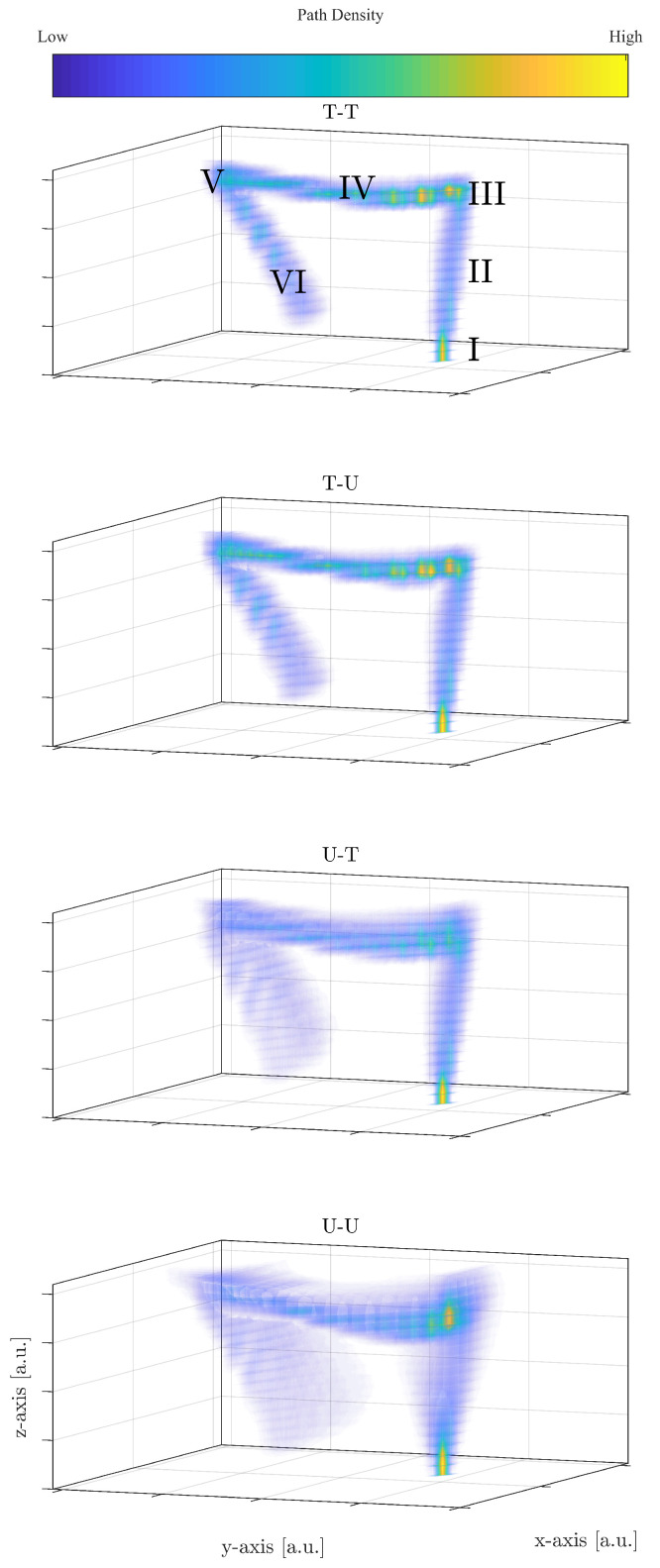
Reconstruction of the large intestine for a random patient, for different prior distributions.

The abbreviations T-T, T-U, U-T, and U-U refer to the combinations of prior distributions of the intestine diameter and endoscopy capsule’s sample frequency, where the first letter refers to the diameter and the second to the sample frequency. T is read as truncated Gaussian distributions and U as uniform distributions. In Figure 5, all reconstructions of the large intestine are shown for a random patient and camera head. The density in Figure 5 is obtained by *N* = 25,000 path reconstructions for this video. A first comparison to Figure 1 illustrates that the proposed model is able to estimate a path that resembles the large intestine. Similar figures can be obtained from the other patients and camera heads.

**Figure 6:**
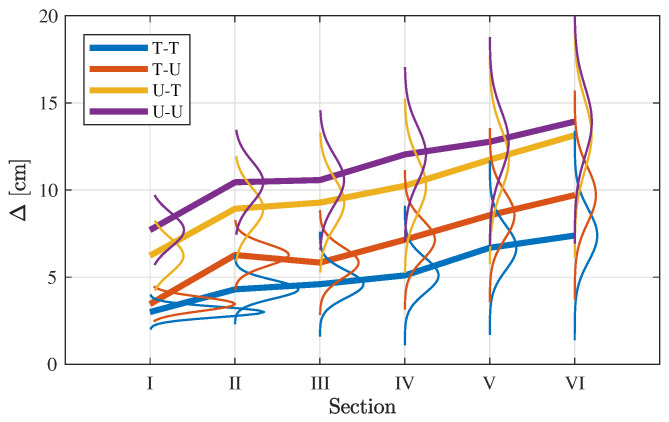
Path difference by large intestine section, S, averaged over all reconstructions for the same video as in Figure 5.

For a more in depth analysis of the obtained estimated paths, let $x_{t,q}={\left[x_{t,q},y_{t,q},z_{t,q}\right]}^{⊤}$ be the vector containing the Cartesian coordinates (in an arbitrary reference frame but fixed for all reconstructions) at time *t* for the *q*th reconstruction. Further, let S=I,II,III,IV,V,VI be for each video ad hoc placed boxes loosely associated with the anus (I), descending colon (II), splenic flexure (III), transverse colon (IV), hepatic flexure (V), and ascending colon (VI), as seen in Figure 1. The path difference between $x_{t,q}\in S$ with respect to any other Cartesian vector $x_{t,w}$ is given by:

(23) $$\begin{array}{ccc} \Delta_{S}=\left|x_{t,q}-x_{t,w}\right|, & \; & q\neq w\in \{1,\ldots ,N\} \end{array}$$

where q,w are an index associated with one of the *N* simulations based on different intestine radii and sample frequencies. Equation (23) is used to extract an assemble of error estimates for all combination of *q* and *w*. The distribution and mean value of Δ are shown in Figure 6 conditioned on each section, S. Overall, Figure 6 shows that the path difference increases as the endoscopy capsule passes more and more sections. Figure 5 and Figure 6 are explained below in greater detail depending on the prior selection:

**T-T**  Figure 5 shows the density of the 25,000 paths estimated, where high densities corresponds to large accumulation of estimated sets of coordinates per unit volume. In the case of T-T, it leads to local patterns of high density. First, the truncated priors estimate a narrow shape of the large intestine compared to the other combinations of priors making the recommended model in the study. This is also seen in the overall path difference, spanning approximative 2–8 cm, well below the other prior combinations (see Figure 6) and in the same order of magnitude as the average radius of the intestine (4.6 cm). Further, as for all cases, the spread of Δ increases as the endoscopy capsules passes the different sections.
  In Section I, the capsules’ path is initialized, which leads the estimated path to be very localized in space resulting in the high density, as seen in Figure 5. In other words, the paths have not yet deviated from one another, apart from small perturbations. As the endoscopy capsule passes the descending colon (II), the individual path appear to spread out more evenly. When reaching the left colic flexure (III), the density in this area is increased, suggesting that the camera spends a period of time in this section in order to complete the turn into the next section.
  When passing along the transverse colon (IV), the paths spread out further, as seen in the distribution of Δ in Figure 6. However, in comparison to Section II, the path along the Transverse Colon has areas of high density. This is explained by the endoscopy capsule spending more time overcoming different segments of the transverse colon. This is an important observation as it might hint to the potential ability of the proposed model recreating local structures in the large intestine.
  Passing Sections V and VI, the local structure is less distinct, while the error increases significantly compared to Section IV with increasing spread of the path difference Δ (see Figure 6).
**T-U**  Adopting a uniform prior for the sample frequency yields the same outcome as in the case of a truncated prior, with the change that the path difference is slightly higher. When comparing the path difference to the one of T-T in Figure 6, the distribution of Δ are comparable in shape to each other, and distinct from the ones using a uniform prior for the radii. Thus, selecting the sample frequency prior has minor impact on the movement estimation.
**U-(T/U)**  Both prior combination do not improve the reconstruction and perform worse by almost a factor of two compared to the favored combinations mentioned above.

**Figure 7:**
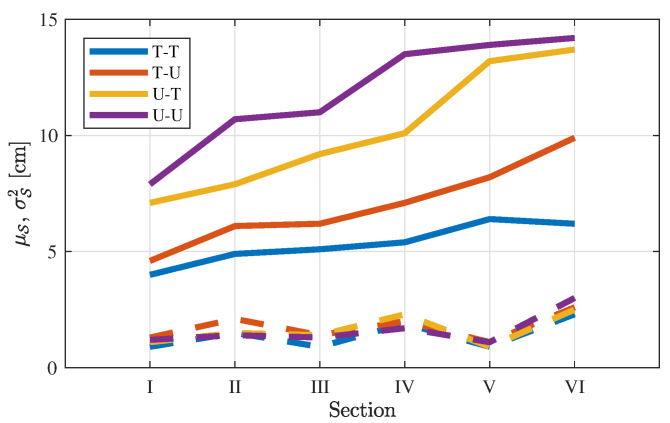
Path difference (solid lines) and variance (dashed lines) by large intestine section, S, averaged over all video.

To evaluate the error propagation further, Figure 7 comprises the descriptive statistics for all 84 videos. For all P=84 videos, the mean value and variance per section are given by:

(24) $$\begin{array}{cc} \mu_{S} & =\frac{1}{P}\sum_{p=1}^{P}E_{p}\left[\Delta_{S}\right] \end{array}$$

(25) $$\begin{array}{cc} \sigma_{S}^{2} & =\frac{1}{P}\sum_{p=1}^{P}E_{p}\left[{\left(\Delta_{S}-E_{p}\left[\Delta_{S}\right]\right)}^{2}\right]. \end{array}$$

In Figure 7, the same increasing trend as Δ in Figure 6 is shown, sorting T-T, T-U, U-T, and U-U in the same way with similar average values. This shows that the propagation of the path difference for the case study of one patient follows the average path difference per section for all patients. The low variance is also evidence for the reproducibility of the reconstructions across patients.

A source of estimation error can be attributed to the quality of the individual frames and the number of feature points extracted from each frame. In the case of the estimated speed, $v_{t+1}$, if the distribution in Equation (6) does not converge sufficiently, especially when the sample size of the distribution is small, the estimate might be wrong. If then poor frames are added, as discussed in Section 2.3, the error will further increase. Table 1 shows the average number of feature points per frame conditioned on the assessed cleanliness and the associated speed.

For increasing speed, and decreasing frame quality, Table 1 shows a decrease in a number of matched feature points between successive frames. This implies fewer samples for the estimation of the movement parameters in Equations (6), (9), and (10). Assuming that more feature points provide better estimates, it is clear that frames of less quality and higher speeds will have an impact on the accuracy of the estimates. Further, through lack of either occurrence or number of feature points, there are no speed estimates for frames of poor quality above 4 mm/s. Ignoring the inferential post-processing of unacceptable frames, the presented model estimates high speeds with many feature points. A high amount of feature points would under normal circumstances (clean frames) be desirable, but justifies here the need to use an inferential predictor, as described in Section 2.3. If this step is omitted, the speed of the endoscopy capsule would have been overestimated—and possibly other parameters would have been estimated erroneously as well. These errors will again be propagated further as the endoscopy capsule advances, compromising the accuracy of the localization.

## 4. Validation

In the absence of a point-wise base line, an inter capsule and expert panel validation and intra endoscopy capsule validation is presented and discussed as an objective evaluation criterion.

### 4.1. Inter Endoscopy Capsule and Expert Panel Validation

Concerning the 84 videos, Sections III and V are annotated by an expert panel, while Section I is chosen as the starting point of the investigation. This allows labeling the remaining Sections II, IV, and VI. With annotated landmarks, the reconstruction, based on the different prior combination, is validated by direct comparison of the individual path segments in the ad hoc boxes and the experts’ labels. Table 2 shows the confusion matrix between the predicted sections and expert labeled sections, for each prior combination. Here, the expert labels are considered *ground truth* for the investigation.

On the diagonal of Table 2, the accuracy of the reconstruction is shown. Across all prior combinations, the accuracy decreases the further away the capsule is from Section I. The T-T model performs the best among the presented prior compilations, with the accuracies 0.92, 0.93, 0.87, 0.82, and 0.76, and U-T the lowest, 0.82, 0.81, 0.72, 0.64, and 0.60. This is in agreement with what has been observed in the path difference, where the error increases with each section. Further, T-T and T-U, as well as U-T and U-U, are closely related to each other, as observed in the path difference. The off-diagonal component of the confusion matrix shows the misclassification of the proposed framework with respect to the experts’ labels. Notice that, in Section VI, the models U-T and U-U show a large spread with contributions in the transverse colon (Section IV). This can be seen in the reconstruction in Figure 5 as part of the estimated paths cross over in other sections. The average accuracy of the T-T model reach reaches 0.86, which is an indication and big encouragement for the potential practical usage of identifying the location and extends of diseases. Furthermore, the misclassification is never larger than the neighboring location.

### 4.2. Intra Endoscopy Capsule Validation

The fact that the endoscopy capsule is equipped with two camera heads, facing opposite directions, presents an opportunity for internal validation. As both camera heads would see the same path, the path estimated by each camera head would be the same up to a minus sign. However, as mentioned above, due to the re-sampling of the time stamps in RAPID™ software, the sample frequency is lost. This loss prohibits a direct time-to-time validation of the movement estimation across the camera heads of a capsule.

**Figure 8:**
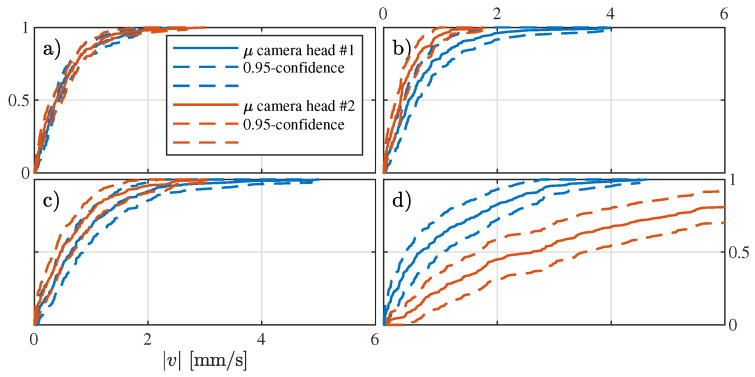
Comparison of the estimated speed cumulative distribution for each camera head for (**a**–**d**) the combinations of priors T-T, T-U, U-T, and U-U, respectively.

Assuming that on average the camera heads perform the same, the estimations will asymptotically be identical for *N* growing large. As an example, Figure 8 provides the cumulative absolute speed distribution for the patient, with *N* = 25,000 path estimations per camera head. In Figure 8a–d, the asymptotic behavior for the different prior combinations is shown. In Figure 8a, high agreement between the camera heads estimations is found, suggesting a robust estimator with high cumulative precision. Note that high precision does not necessarily imply accuracy. Figure 8b,c shows agreement between the two camera heads within their 95% confidence interval. In contrast, the model based on only uniform priors (see Figure 8d) shows no agreement between the camera heads.

## 5. Discussions and Conclusions

In this work, we present a movement estimation model for capsule endoscopy based on feature point tracking in successive frames. Furthermore, to reconstruct the large intestine as viewed by the endoscopic capsule, a tracking and localization model is also proposed. This demonstration contributes to aid physicians in assessing the location of an endoscopic capsule based on the retrieved videos, without implementing invasive measures. This carries especially clinical relevance in terms of identification of complete visualizations and thereby location of significant findings and abnormalities such as colorectal polyps, while revealing the anatomy of individuals undergoing the examination of the colonic segment. This knowledge impacts determining further treatment of the patients, such as type of diagnostic, therapeutic, or bowel preparation procedure.

Besides introducing different realizations of reconstructed paths of an endoscopic capsule, namely prior selection, each model’s path difference was evaluated and compared against each other. This showed that the choice of priors on sample frequency has less impact than that of the priors on the radius of large intestine. We further pointed out and thoroughly discussed a list of challenges associated with the visual based models, concluding that error propagation and frame cleanliness can contribute to the estimation error. Nonetheless, the T-T model of this study achieves an average accuracy of 0.86, with misclassification error not larger than the neighboring section throughout the large intestine.

Applications of this study will focus on evaluating the feasibility in clinical use, especially with respect to aiding in localization of significant findings such as colorectal polyps in combination with polyp detection algorithms and estimation of complete and incomplete investigations. Future research on the localization will address the remaining challenge of validation and error propagation. Other areas to which this work can be extended to are the reconstruction of the small intestine. One way of facilitating this could be through inclusion of other visual-based processing techniques. As neural networks have become better and better in image processing, smart image processing could pave the way in the near future. Deep learning and smart image processing in capsule endoscopy have recently attracted attention in texture classification [43], polyp, abnormality detection and segmentation [44,45,46], and localization [31,32].

## Figures and Tables

**Table 1:** Average number of feature points per frame conditioned on the cleanliness and estimated speed.

Cleanliness	Avg. # of Feature Points per Frame				
per Frame	Speed Intervals [mm/s]				
	|[0,2[|	|[2,4[|	|[4,6[|	|[6,8[|	|[8,∞)|
Good (0)	131	98	32	29	24
Fair (1)	90	76	25	25	21
Poor (2)	54	28	-	-	-
Unacceptable (3)	-	-	-	186	210

**Table 2:** Confusion matrix of predicted and expert labeled sections.

True (Expert) Label																						
	Section		II				III				IV				V				VI			
		Prior	T-T	T-U	U-T	U-U	T-T	T-U	U-T	U-U	T-T	T-U	U-T	U-U	T-T	T-U	U-T	U-U	T-T	T-U	U-T	U-U
Predicted Label	II	T-T	0.92				0.02															
		T-U		0.91				0.03														
		U-T			0.86				0.05													
		U-U				0.82				0.05												
	III	T-T	0.08				0.93				0.07											
		T-U		0.09				0.92				0.07										
		U-T			0.14				0.85				0.09									
		U-U				0.18				0.81				0.09								
	IV	T-T					0.05				0.87				0.09							
		T-U						0.05				0.85				0.09						
		U-T							0.10				0.76				0.11				0.03	
		U-U								0.14				0.72				0.12				0.07
	V	T-T									0.06				0.82				0.24			
		T-U										0.08				0.78				0.26		
		U-T											0.15				0.65				0.33	
		U-U												0.19				0.63				0.33
	VI	T-T													0.09				0.76			
		T-U														0.13				0.74		
		U-T															0.24				0.63	
		U-U																0.25				0.60

## Data Availability

The data cannot be shared with other organizations.
